# Supplementary material for: Bioinspired Swimming Robots with 3D Biomimetic Shark Denticle Structures for Controlled Marangoni Propulsion
Source: Biomimetics (Basel). 2025 Jul 22;10(8):479. doi: 10.3390/biomimetics10080479 (PMC12383345; doi:10.3390/biomimetics10080479)
Supplement: Supplementary file 1 [file biomimetics-10-00479-s001.zip › biomimetics-3711786-supplementary.pdf]

Bioinspired Swimming Robots with 3D Biomimetic Shark Denticle Structures for Controlled Marangoni Propulsion

Kang Yang [1,2], Chengming Wang [1,2], Lei Jiang [1,2], Ruochen Fang [3,*] and Zhichao Dong [1,2,*]

[1] CAS Key Laboratory of Bio-inspired Materials and Interfacial Science, Technical Institute of Physics and Chemistry, Chinese Academy of Sciences, Beijing 100190, China

[2] School of Future Technology, University of Chinese Academy of Sciences, Beijing 100049, China

[3] International Institute for Interdisciplinary and Frontiers, Beihang University, Beijing 100191, China

*Corresponding Authors: Zhichao Dong, E-mail: dongzhichao@mail.ipc.ac.cn

Ruochen Fang, E-mail: fangrc@buaa.edu.cn

Supplementary information

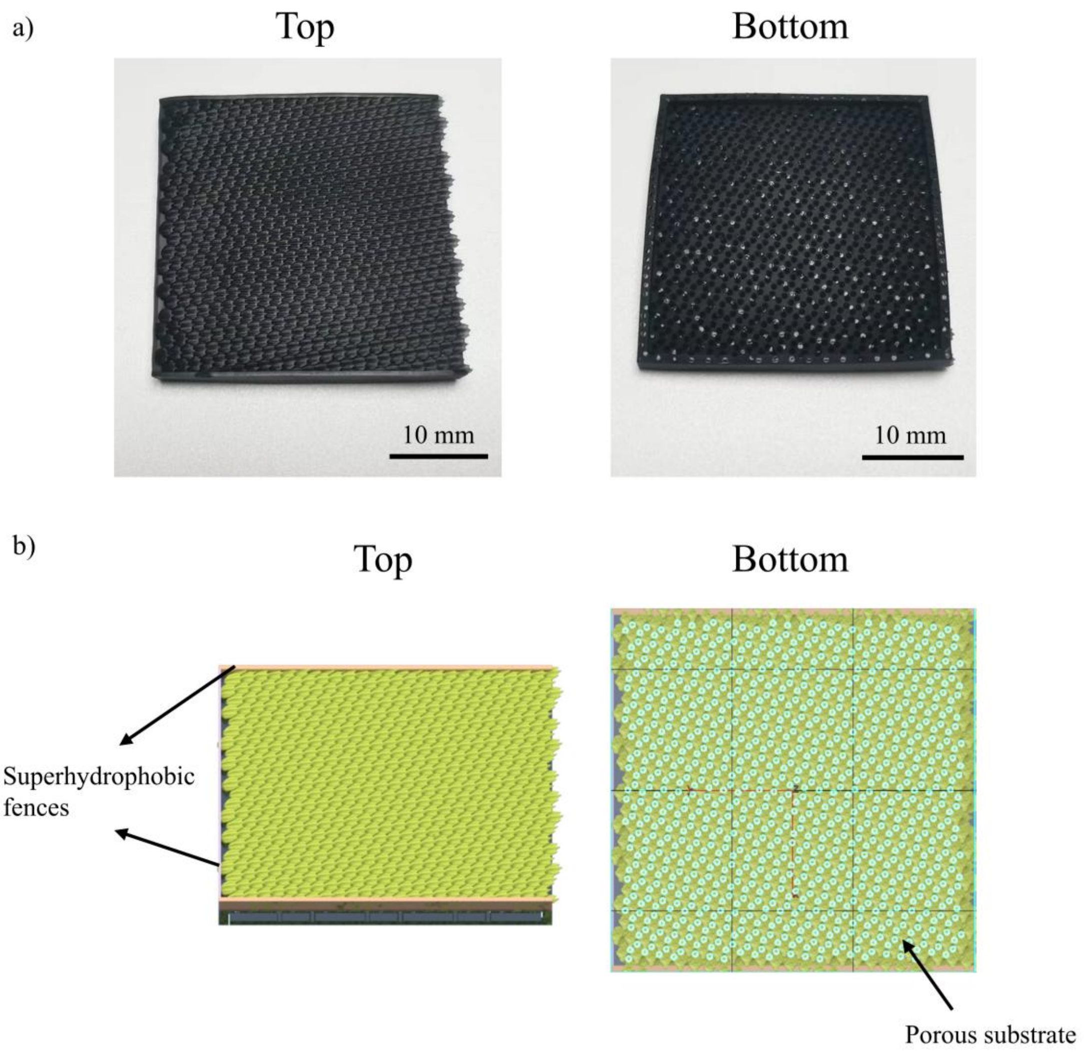

Figure S1. (**a**) Photography of the top and bottom of the 3D-printed Marangoni swimmer with staggered and overlapped denticle arrays. (**b**) 3D design rendering of the top and bottom of the Marangoni swimmer with staggered and overlapped denticle arrays, showing superhydrophobic fences along sides of the swimmer and the porous substrate in the bottom.

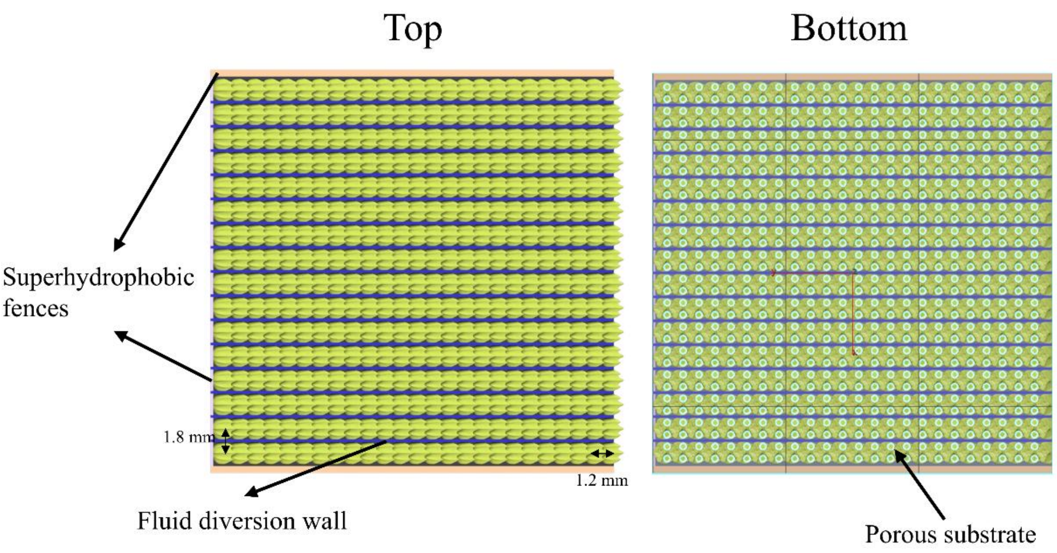

Figure S2. 3D design rendering of the top and bottom of the Marangoni swimmer with a linear denticles arrangement, showing superhydrophobic fences along sides of the swimmer, diversion walls between every two columns of denticles, and the porous substrate in the bottom.

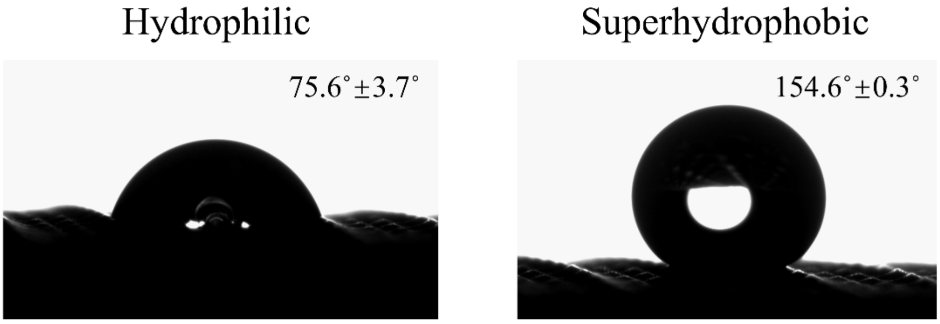

Figure S3. Contact angles for hydrophilic and superhydrophobic denticles of the 3D-printed Marangoni swimmer.

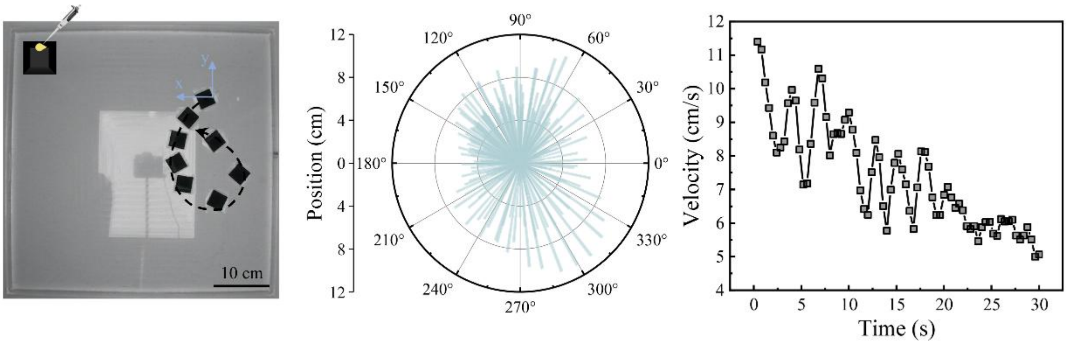

Figure S4. Self-propelled Marangoni swimmer with staggered and overlapped denticle arrays exhibiting certain counterclockwise rotational motion when HFIP is introduced into all through-holes in the right half of the swimmer.

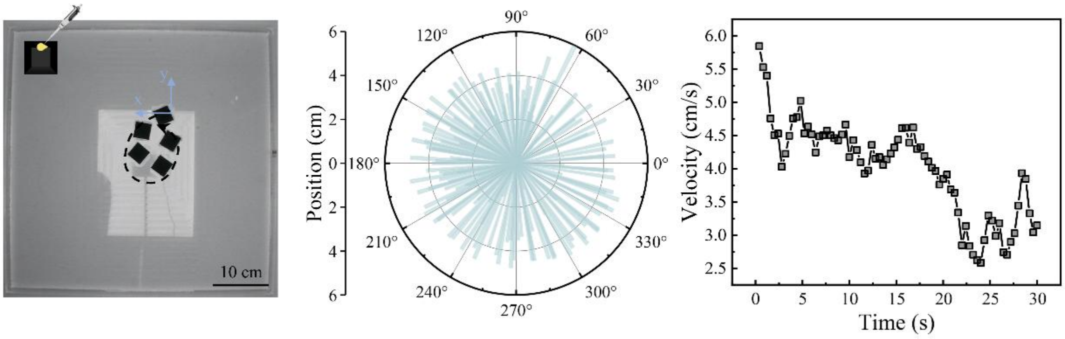

Figure S5. Self-propelled Marangoni swimmer with a linear denticle arrangement exhibiting continuous and regular counterclockwise rotation when HFIP is introduced into the right column of the swimmer.

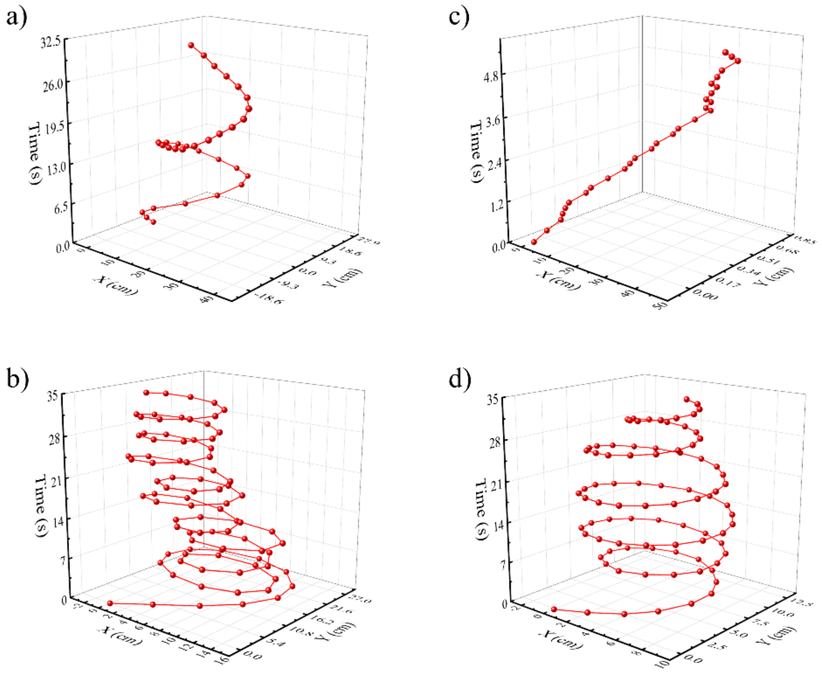

Figure S6. The rotation trace of self-propelled Marangoni swimmer at different times. (**a**) Swimmer with staggered and overlapped denticle arrays exhibiting unstable trajectories when HFIP is introduced into the central through-holes on the bottom of the swimmer. (**b**) Swimmer with staggered and overlapped denticle arrays exhibiting certain clockwise rotational motion when HFIP is introduced into all through-holes in the left half of the swimmer. (**c**) Swimmer with a linear denticle arrangement exhibiting linear forward motion when HFIP is introduced into the central holes at the bottom of the swimmer. (**d**) Swimmer with a linear denticle arrangement exhibiting continuous and regular clockwise rotation when HFIP is introduced into the left column of the swimmer.

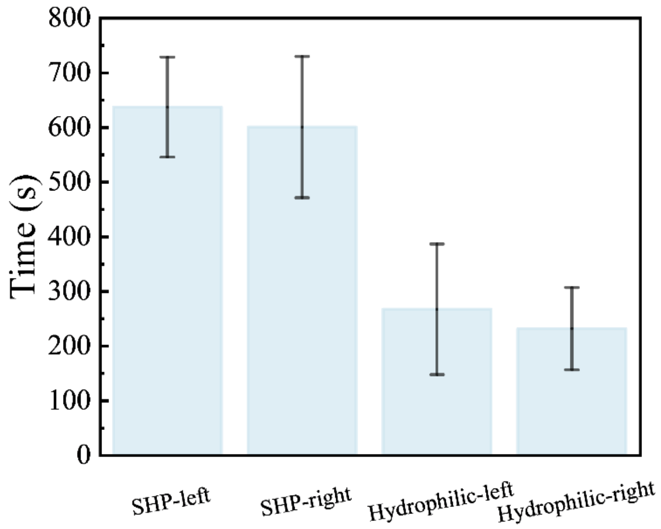

Figure S7. Cruise duration of the hydrophilic and superhydrophobic self-propelled Marangoni swimmer (HFIP into the left or right column).

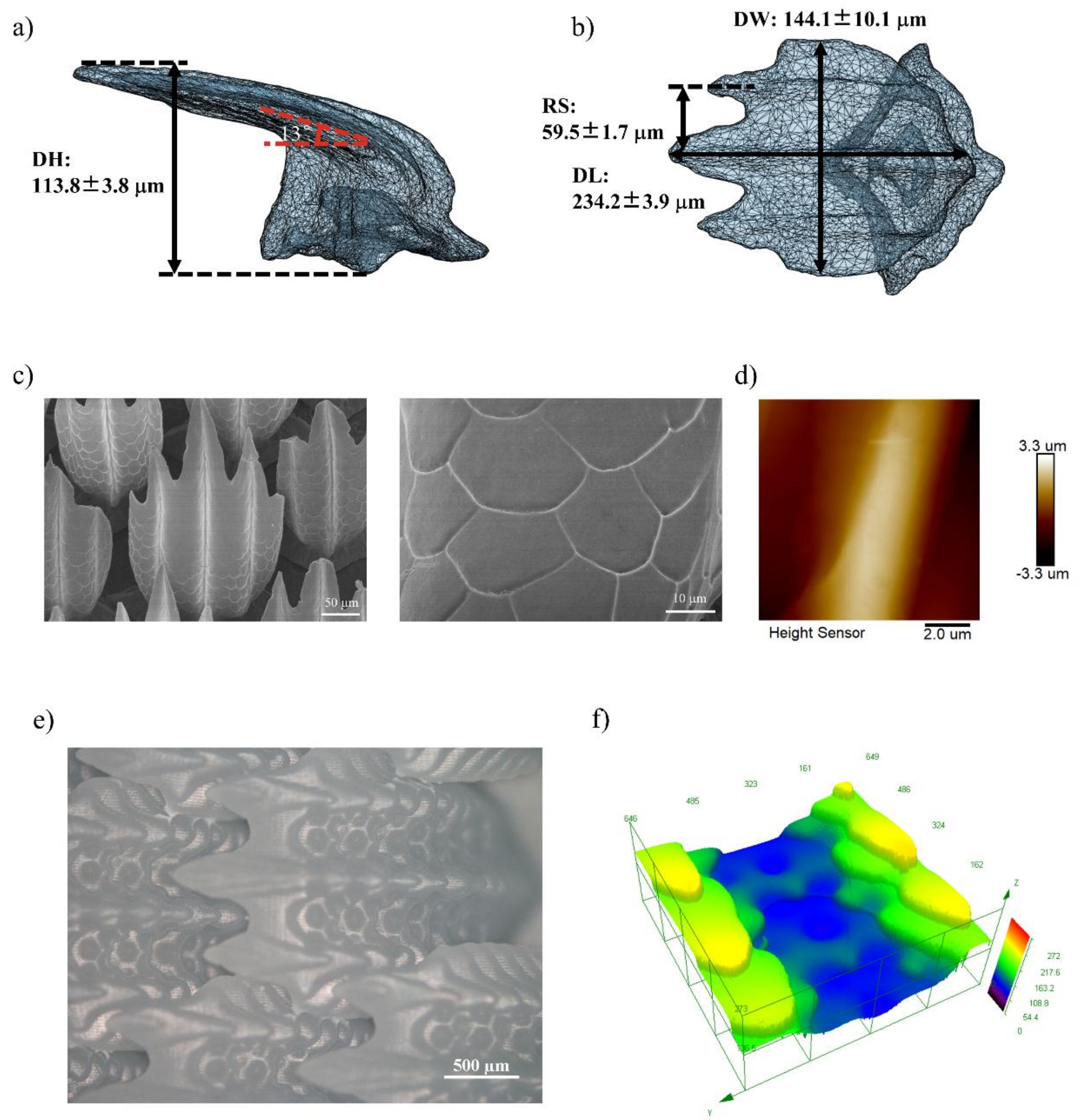

Figure S8. Characterization of Pacific spadenose shark (*Scoliodon macrorhynchos*) denticles. (**a, b**) Side view (**a**) and top view (**b**) of 3D characteristics of a representative denticle of a Pacific spadenose shark. (**c**) SEM image of shark denticles. (**d**) Atomic force microscopy image of the hexagonal pit structure. (**e**) Optical image of 3D-printed Pacific spadenose shark denticles. (**f**) Surface morphology of 3D-printed Pacific spadenose shark denticles.

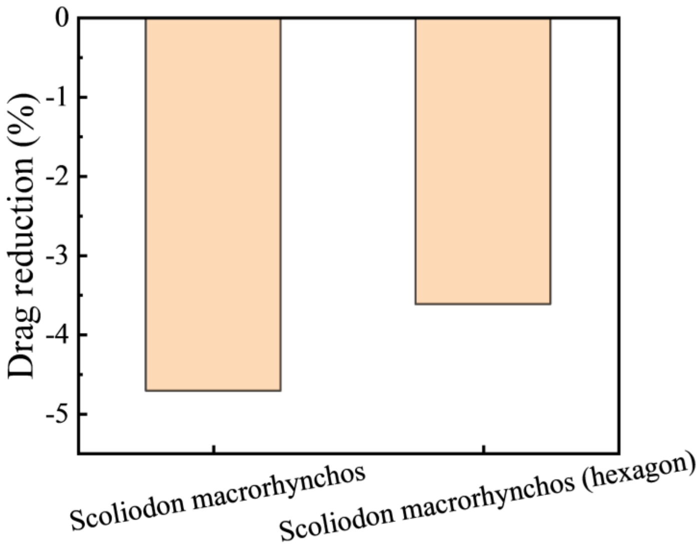

Fig. S9. Drag reduction performance of the Pacific spadenose shark denticles (with and without hexagonal structures) in closed circulating water tunnel experiment at a flow rate of 2m s^{-1}.

Table S1. Comparison of Shark Drag Reduction Methods and Results Across Studies.

	Fluid environments	Testing conditions	Drag reduction method	Structures and components involved in drag reduction	Propulsion mechanisms (Results)
Ref [31]	Turbulent	Wind tunnel	Passive drag reduction	Riblet and denticle structure	1) Riblet structure reducing streamwise vortices. 2) vortex generators which enhance mixing and keep the flow attached. 3) Passive denticles bristling inhibits flow separation. 4) streak cancellation due to pressure difference.
Ref [32]	\	\	\	Denticle structure	1) Passive denticle erection to control flow separation. 2) Mucus mechanism is unknown.
Ref [33]	Turbulent	Theoretical (viscous flow) calculations	Passive drag reduction	Riblet structure with different shape	Protrusion height theory (impede the spanwise movement of streamwise vortices).
Ref [34]	Turbulent	Theoretical calculations and electrolytic experiments	Passive drag reduction	Riblet structure with different shape	1) Shear stress reduction mechanisms (involve streamwise vortices). 2) Delay of flow separation.
Ref [35]	Turbulent	Oil channel	Passive drag reduction	2D and 3D Riblet structure	Protrusion height theory.
Ref [36]	Turbulent	Oil channel	Passive drag reduction	Riblet structure	1) Slit ejection mechanism. 2) Protrusion height theory.
Ref [37]	Turbulent	Oil channel	Passive drag reduction	Shark skin replica	\
Ref [38]	Turbulent	Wind tunnel	Passive drag reduction	Riblet structure with different shape	\
Ref [39]	Turbulent	Wind tunnel	Passive drag reduction	Riblet structure with different shape	\
Ref [40]	Turbulent	Flapping foil in water flow tank	Passive drag reduction	3D-printed denticles	The formation of the leading edge vortex (LEV), which has a significant impact on lift

					force production and leading edge suction force.
Ref [41]	Turbulent	Flapping foil in water flow tank	Passive drag reduction	3D-printed denticles	Same as above
Ref [42]	Turbulent	Water tunnel	Passive drag reduction	Real shark skin (denticles)	Passive flow separation control.
Ref [43]	\	Water tank	Passive drag reduction	3D-printed denticles	The subsurface among denticle flows could be minimal.
Ref [44]	Turbulent	Microfluidic chamber	Passive drag reduction	Real shark skin (denticles)	Small eddies, and recirculation bubbles behind the natural dermal denticles.
Ref [45]	Turbulent	Direct numerical simulation	Passive drag reduction	Denticle structure	Reversed flow in the underneath cavity generates significant thrust under adverse pressure (drag reduction 4%).
This work	Disturbed laminar flow near a free surface	Water tank	Active drag reduction	3D-printed denticles and surfactants	Marangoni-driven propulsion arises from surface tension gradients induced by surfactants; with higher surfactant concentrations and lower surface tension enhancing propulsion performance.

Table S2. Comparison of Chemical Fuels: Surface Tension and Propulsion Velocity of the Marangoni-Driven Swimmer.

Chemical fuel	Surface tension (mN/m)	Propulsion velocity (cm/s)
Hexafluoroisopropanol (HFIP)	14.53	8.63
Isopropanol (IPA)	21.70	4.14
N-propanol	23.78	2.82
Ethanol (EtOH)	22.39	2.25
Dimethyl sulfoxide (DMSO)	43.60	1.17
